# Fine-Scale Genetic Structure Arises during Range Expansion of an Invasive Gecko

**DOI:** 10.1371/journal.pone.0026258

**Published:** 2011-10-28

**Authors:** Kristen Harfmann Short, Kenneth Petren

**Affiliations:** Department of Biological Sciences, University of Cincinnati, Cincinnati, Ohio, United States of America,; Smithsonian Institution National Zoological Park, United States of America

## Abstract

Processes of range expansion are increasingly important in light of current concerns about invasive species and range shifts due to climate change. Theoretical studies suggest that genetic structuring may occur during range expansion. Ephemeral genetic structure can have important evolutionary implications, such as propagating genetic changes along the wave front of expansion, yet few studies have shown evidence of such structure. We tested the hypothesis that genetic structure arises during range expansion in *Hemidactylus mabouia*, a nocturnal African gecko recently introduced to Florida, USA. Twelve highly variable microsatellite loci were used to screen 418 individuals collected from 43 locations from four sampling sites across Florida, representing a gradient from earlier (∼1990s) to very recent colonization. We found earlier colonized locations had little detectable genetic structure and higher allelic richness than more recently colonized locations. Genetic structuring was pronounced among locations at spatial scales of tens to hundreds of meters near the leading edge of range expansion. Despite the rapid pace of range expansion in this introduced gecko, dispersal is limited among many suitable habitat patches. Fine-scale genetic structure is likely the result of founder effects during colonization of suitable habitat patches. It may be obscured over time and by scale-dependent modes of dispersal. Further studies are needed to determine if such genetic structure affects adaptation and trait evolution in range expansions and range shifts.

## Introduction

Genetic structure often arises as the result of restricted gene flow and genetic drift among populations over relatively long periods of time [1]. During range expansion, however, genetic structure can arise quickly. At first this may seem counterintuitive because expanding populations are typically large, but structure arises primarily at the leading edge of the expansion where population sizes can be quite small [2], [3]. When dispersal is limited in a patchy environment, colonization of new populations at the leading edge of range expansion can create genetic structure reflecting small founding populations that carry only a subset of genetic diversity and are isolated from other sources of migrants [2], [3]. Over time, gene flow among populations may erode structure [4], [5], but even temporary genetic structure at the leading edge can have lasting effects on the evolutionary trajectory of an expanding population. If leading edge populations serve as sources of migrants for subsequent colonizations, any changes will be propagated across the landscape through successive colonizations [6]. The potential evolutionary consequences of such structure could include inbreeding effects, limited response to selection or even enhanced response to selection due to mutation surfing [7], [8], [9]. Understanding the dynamics at the leading edge of range expansion is critically important, because range expansions associated with biological invasions are increasing, and because range shifts, which require expansion in at least one direction, are expected to occur broadly due to climate change [10], [11].

Despite the potential importance of genetic structure at the leading edge of range expansions, very few empirical studies have investigated it [5], [12], [13]. However, this lack of empirical data does not necessarily indicate that genetic structure plays no role in range expansions in nature. Instead, the fine spatial and temporal scales over which genetic structure can arise make it more difficult to detect than genetic structure in large, stable populations. Indeed, while several theoretical studies suggest that genetic structure should arise during colonization of new populations and eventually fade as gene flow among them increases [3], [4], most empirical studies of genetic structure during range expansion have been conducted only at relatively large spatial and temporal scales [14], [15]. Patterns may be qualitatively different at finer scales, where modes of dispersal and the effect on genetic structuring may differ [16], [17], [18]. In addition, the expectation that fine-scale genetic patterns at the leading edge of expansion will erode over time [5] implies that it will be difficult to detect structure over large temporal scales. A first step toward understanding the importance of genetic structuring during range expansion is to understand where and when it arises in natural populations. This can be achieved with sampling strategies, genetic techniques, and model systems that are especially suitable for revealing fine-scale genetic structure.

Invasive species are ideal systems for investigating dynamics of range expansion [19]. A number of landscape-scale studies have revealed patterns of genetic structure in expanding invasive populations [15], [20]. However, fine-scale patterns are especially likely to differ from larger scale patterns in invasive populations because large-scale patterns may reflect mass (human-mediated) dispersal, while finer scale patterns may reflect natural dispersal [16], [17], [18], [21]. Therefore, even in populations that appear to have very little landscape-scale genetic structure, fine-scale patterns may reveal very different processes that have important evolutionary implications. Invasive populations also represent natural experiments that can be used to better understand how genetic structure changes over time as range expansion proceeds. Genetic structure can be quantified among populations at the leading edge of invasion and then compared to structure among populations nearer the center of the range. Knowledge that the invasion is ongoing limits the possibility that any structure revealed is the product of longer-term processes, and it can therefore be attributed to the dynamics of the expansion process.

We used the ongoing invasion of the tropical house gecko *Hemidactylus mabouia* as a model system to investigate the fine-scale genetic patterns that arise during range expansion. *Hemidactylus mabouia* is native to Africa [22], and was first recorded in Miami, Florida in the early 1990s [23]. It has subsequently spread westward and northward throughout the state, primarily occupying human structures and gardens where many invasive species thrive [24]. Because populations range in age from 20–30 years since colonization in the South to very recently colonized (1–2 yrs. ago) in the North [25]. The invasion occurs across a patchy landscape. House geckos away from their native tropical forests are generally confined to human structures because most buildings provide shelter during the day, lights and eves to collect insects, and flat walls that increase foraging efficiency, as has been shown by surveys across the Pacific [26] and replicated field experiments [27], [28], [29]. In Florida, geckos are rarely observed away from human structures, neighboring buildings often differ dramatically in the density of the invasive and prior resident gecko species, and relative abundance changes predictably over time through colonization and population growth [25]. Genetic differentiation among major metropolitan centers across Florida is, not surprisingly, very limited, given the rapid pace of spread and the likely role that the transport of human goods has played in aiding their dispersal (average *F_st_/θ* = 0.06) [30]. These features make this system suitable to address the question of whether fine-scale genetic patterns are present in a rapidly expanding population, as predicted by theory.

We tested the hypothesis that fine-scale genetic structure arises during the colonization of new locations in Florida by *H. mabouia*. The likely cause of increased structure at these brief time scales is the subsampling of genetic diversity during colonization and founding events, so we also tested for the predicted loss of genetic diversity in recently colonized locations. While limited gene flow should generate population structure initially, even low levels of subsequent gene flow among locations will erode the signature of colonization. Therefore, we also tested the hypothesis that genetic structure will be most pronounced at the leading edge of invasion by comparing the genetic structure of recently colonized sites with that in Miami, the source of the Florida invasion. These tests were conducted in 43 locations in four sample sites across Florida using genetic variation at twelve highly variable microsatellite loci.

## Materials and Methods

### Ethics Statement

All tissue collection for this study was approved by the Institutional Animal Care and Use Committee at the University of Cincinnati under protocol 06-06-01-01, and all efforts were made to minimize animal suffering.

### Sample Collection and Genotyping

*Hemidactylus mabouia* is a nocturnal, sexually reproducing, insectivore that can be found commonly on human structures. Buildings represent patches because the habitat between buildings (grass, sidewalks, pavement) is generally unsuitable and likely limits dispersal. We collected 418 gecko tissue samples (tail tips) from 43 locations (buildings) in Florida (Table S1). We chose four main sites with similar architecture and a similar abundance of suitable and accessible structures with high gecko densities. The structures at these four sites are similar to those throughout Florida and they are easily accessible. All four sites have intervening habitat that is unsuitable for geckos as judged by their absence. Sidewalks and lawns that make up intervening habitat at the two university sites could, in principle, be occupied by geckos at night, but they are extremely unsuitable during the day because of the lack of hides, strong sun and frequent heavy rain. Similarly, the shrubs and trees in the intervening habitat of the Everglades and Fort De Soto do not have detectable gecko densities except immediately adjacent to buildings, presumably due to low densities of catchable insects, complex habitat structure [29] and a lack of secure hides (e.g. peeling bark). Palm trees with hanging dead fronds for hides may support low densities of geckos, but they are generally sparsely distributed.

As *H. mabouia* has colonized Florida, it has displaced other introduced gecko species. The relative abundance of *H. mabouia* compared to other species in recent censuses provides an indication of colonization time, with higher *H. mabouia* abundance reflecting earlier colonization. During censuses from 1998–2009 [24], [25], no other gecko species were recorded at the University of Miami (M), near the site of introduction, so this site was the earliest colonized. However, *H. mabouia* abundances were lower at other sites, reflecting more recent colonization: Everglades National Park (E; ∼90% in 1998, 100% in 2003), Fort De Soto Park (D; ∼90% in 2009) and Florida Institute of Technology (F or F.I.T.; 60% in 2004; most recently colonized).

Geckos were captured by hand from March–September 2009 and the location of each individual was noted with a handheld GPS (Garmin). Tail tissue samples were collected and stored in 70% ethanol. We amplified 12 microsatellite loci developed for *H. mabouia* using multiplex PCR with four loci in each reaction [31]. Fragment analyses were conducted on an ABI 3730xl DNA analyzer with -500 LIZ size standard at the Cornell Biotechnology Resource Center. Allele calls were verified by eye in Genemapper 3.7 (Applied Biosystems).

### Population Structure and Gene Flow

To determine the degree of population differentiation, we estimated *F_st_* by calculating *θ*, which accounts for small and unequal sample sizes, in gda v.1.1 [32], [33]. Confidence intervals (95% CI) on *θ* were calculated by bootstrapping across loci in gda and used to assess overall levels of structure within sites. We tested for differences in pairwise *θ* values among locations using exact tests in genepop [34] and among sites using 10,000 permutations in fstat [35]. Although *θ* accounts for small and unequal sample sizes, we further explored the effect of sample size differences on *θ* with resampling down to a size of 3 individuals. We used analysis of molecular variance (amova) to partition genetic variance among hierarchical levels [36], and tested for isolation by distance among locations within sample sites with Mantel tests conducted in GenAlEx [37]. We also conducted tests for significant spatial autocorrelation using variable distance classes in GenAlEx [38], with 1000 permutations and 1000 bootstraps to generate 95% confidence intervals.

We used the Bayesian clustering program structure v.2.3.1 [39], [40], [41] to cluster individuals according to Hardy Weinberg and linkage equilibrium. We used the admixture model, correlated allele frequencies, and sample location information to conduct simulations with burn-in of 25,000, followed by 100,000 iterations of Markov Chain Monte Carlo, and 10 simulations at each K. Subsampling our data set and repeating structure analyses produced no qualitative change in the results, so sample size differences among sites can be ruled out as a factor that accounts for clustering differences. To incorporate geographic information into our analyses of population structure, we also used spatial Bayesian inference in geneland [42] to cluster individuals within sample sites. We ran simulations with no spatial uncertainty and a burn-in of 10,000, followed by 90,000 iterations of MCMC.

We used MCMC simulations to assess the relative likelihoods of a migration-drift equilibrium (gene flow) model versus a nonequilibrium drift model. We used 2MOD [43] with a burn-in of 10,000, followed by 90,000 MCMC iterations, and probabilities for each model were calculated from the proportion of runs supporting each model. We derived the Bayes factor from the ratio of runs supporting each model. This approach mainly relies on the assumption that mutation is not an important factor in creating novel alleles, relative to migration, which seems appropriate for the short time of a recent introduction.

### Genetic Diversity

Tests for null alleles were conducted in microchecker v. 2.2.3 [44]. Summary statistics such as expected and observed heterozygosity for each location were conducted in GenAlEx 6.1 [31]. Exact tests for Hardy Weinberg equilibrium were done in genepop on the Web [45], and one-tailed probabilities for heterozygote deficit were corrected for multiple comparisons with the sequential Bonferroni correction. Tests for linkage equilibrium were also conducted in genepop on the Web.

Allelic richness was calculated using rarefaction to account for differences in sample size among locations in fstat v.2.9.3.2 [34]. We calculated allelic richness at individual locations across all four sample sites (standardized to 3 individuals), and also pooled data within sample sites (standardized to 59 individuals). We tested for significant differences in allelic richness at individual locations among sample sites using 10,000 permutations in fstat. To test for differences in allelic richness among sites (pooling locations within sites), we used a Wilcoxon sign-rank test. Significance tests were conducted in JMP 7.0, with alpha of 0.05.

We tested for bottlenecks on individual buildings with bottleneck v.1.2.02 [46]. We conducted simulations assuming the two-phase model of microsatellite mutation with 95% stepwise mutations. We report results of the two-tailed Wilcoxon sign-rank test because it is the most powerful test that is suitable for fewer than 20 loci [47].

## Results

We sampled 124 individuals from 12 locations at the University of Miami (M1–M12; max. distance 1.52 km), 140 individuals from 12 locations in the Everglades National Park (E1–E12; max. distance 48 km), 90 individuals from 9 locations at Fort De Soto (D1–D9; max. distance 4.82 km), and 64 individuals from 10 locations at F.I.T. (F1–F10; max. distance 0.64 km; Table 1). There was some evidence suggesting the presence of null alleles at three loci, but exclusion of these loci did not produce different results. There was no evidence for linkage disequilibrium within any of the four sites. Some locations at each site (11 total, 25%) showed evidence of Hardy Weinberg heterozygote deficit (Table 1).

**Table 1:** Summary of genetic variation across all locations at four sites.^1^

Sample Sites and Buildings	*N*	*N_A_*	*A_R_*	*H_E_*	*H_O_*
**University of Miami**	**124**	**5.6**	**5.01/2.72**	**0.59**	**0.53**
M1	10	3.3	2.59	0.52	0.54
M2	10	4.0	2.80	0.55	0.55
M3	10	3.5	2.62	0.54	0.48
M4	11	4.0	2.83	0.58	0.52*
M5	9	3.5	2.66	0.54	0.45*
M6	5	3.3	2.70	0.49	0.42
M7	11	3.8	2.66	0.54	0.61
M8	12	3.8	2.74	0.59	0.57
M9	8	3.4	2.81	0.60	0.50*
M10	13	4.3	2.70	0.54	0.53
M11	12	4.2	2.88	0.59	0.60
M12	13	3.8	2.63	0.54	0.51
**Everglades National Park**	**140**	**5.4**	**4.95/2.66**	**0.60**	**0.51**
E1	17	3.7	2.46	0.51	0.47*
E2	10	3.7	2.68	0.53	0.45*
E3	13	3.5	2.73	0.59	0.55
E4	14	3.9	2.83	0.60	0.63
E5	16	3.8	2.54	0.53	0.48*
E6	5	3.2	2.66	0.53	0.55
E7	8	3.1	2.46	0.53	0.58
E8	13	3.6	2.51	0.50	0.41*
E9	13	4.2	2.74	0.56	0.51
E10	12	3.9	2.82	0.59	0.59
E11	14	4.3	2.63	0.52	0.48
E12	5	3.3	2.81	0.55	0.52
**Fort De Soto Campground**	**90**	**5.1**	**4.90/2.36**	**0.52**	**0.41**
D1	10	3.1	2.23	0.43	0.34
D2	11	2.8	2.01	0.36	0.34
D3	11	3.3	2.45	0.50	0.39*
D4	11	3.4	2.41	0.47	0.44
D5	12	3.3	2.49	0.53	0.45*
D6	11	3.8	2.51	0.49	0.47
D7	10	2.7	2.10	0.40	0.38
D8	8	3.4	2.58	0.49	0.45
D9	6	2.9	2.47	0.48	0.50
**Florida Institute of Technology**	**64**	**4.8**	**4.73/2.20**	**0.53**	**0.43**
F1	7	2.9	2.30	0.43	0.34*
F2	6	2.8	2.43	0.50	0.49
F3	3	2.2	2.17	0.36	0.38
F4	11	3.7	2.69	0.57	0.49*
F5	7	2.8	2.26	0.42	0.46
F6	10	3.2	2.43	0.48	0.46
F7	5	2.1	1.91	0.33	0.37
F8	3	1.8	1.83	0.30	0.33
F9	6	2.2	1.94	0.35	0.43
F10	6	2.3	2.00	0.35	0.40

1 *N* is sample size, *N_A_* is number of alleles, *A_R_* is allelic richness, *H_E_* is expected heterozygosity, and *H_o_* is observed heterozygosity. Bold numbers indicate values for entire populations when locations are pooled; they are NOT averages among locations, except in the following case: for allelic richness, the first bold number represents pooled locations, while the second bold number after the slash represents location averages. Starred (*) values for observed heterozygosity reflect significant heterozygote deficits at those locations.

### Population Structure and Gene Flow

Near the origin of invasion at the University of Miami there was little genetic differentiation among locations (mean *θ* = 0.020), it was only marginally significantly different from zero (lower CI = 0.000), and 10.6% of all pairwise comparisons were significant (Table 2). In contrast, the three more recently colonized sites showed greater levels of differentiation (Everglades: *θ* = 0.055; lower CI>0; 59.1% of pairwise comparisons were significant; Fort De Soto: *θ* = 0.078; 75% pairs significant; F.I.T.: *θ* = 0.108, lower CI>0; 51.1% pairs significant). Comparisons among sites showed that Miami was significantly less differentiated compared to F.I.T. (*P* = 0.002) and marginally so from Fort De Soto (*P* = 0.053), while the Everglades was also marginally less differentiated than F.I.T. (*P* = 0.063). Resampling simulations confirmed that slightly lower sample sizes at Fort De Soto and F.I.T. did not explain their higher *θ* values (results not shown).

**Table 2:** Pairwise theta values among locations at each sample site.^1^

	M1	M2	M3	M4	M5	M6	M7	M8	M9	M10	M11	M12
M1												
M2	0.014											
M3	0.011	0.050										
M4	0.005	0.036	0.017									
M5	0.027	0.020	0.028	0.018								
M6	0.003	0.003	0.019	0.012	0.034							
M7	0.033	0.028	0.041	0.038	0.035	0.039						
M8	**0.037**	**0.042**	0.021	**0.039**	0.035	0.029	**0.035**					
M9	0.022	0.015	0.015	−0.005	0.011	−0.005	0.014	0.004				
M10	0.002	0.017	0.036	**0.055**	**0.066**	−0.001	0.021	**0.038**	0.030			
M11	0.006	0.006	0.014	0.026	0.023	−0.007	0.005	0.001	−0.009	0.005		
M12	0.005	0.027	0.025	0.022	0.017	0.003	0.007	0.021	0.005	0.004	0.001	

	E1	E2	E3	E4	E5	E6	E7	E8	E9	E10	E11	E12
E1												
E2	**0.043**											
E3	**0.069**	**0.039**										
E4	**0.043**	0.017	**0.031**									
E5	**0.104**	**0.101**	**0.081**	**0.108**								
E6	0.027	0.049	**0.058**	0.044	0.028							
E7	**0.098**	**0.085**	**0.088**	**0.084**	**0.056**	0.048						
E8	**0.080**	**0.030**	**0.072**	**0.050**	**0.051**	**0.043**	**0.110**					
E9	0.017	0.023	0.015	0.028	**0.069**	0.023	**0.060**	**0.047**				
E10	**0.035**	**0.037**	**0.050**	**0.047**	**0.087**	0.029	**0.075**	**0.066**	0.013			
E11	**0.064**	0.024	**0.087**	**0.045**	**0.120**	0.074	**0.115**	**0.066**	0.038	**0.059**		
E12	−0.022	−0.020	0.018	0.001	0.021	0.002	0.045	−0.005	−0.012	−0.012	0.020	

	D1	D2	D3	D4	D5	D6	D7	D8	D9			
D1												
D2	**0.090**											
D3	**0.149**	**0.177**										
D4	0.026	**0.062**	**0.073**									
D5	0.029	**0.088**	**0.074**	0.022								
D6	**0.058**	**0.080**	**0.053**	0.014	**0.038**							
D7	**0.090**	−0.020	**0.118**	**0.060**	**0.080**	**0.057**						
D8	**0.174**	**0.197**	**0.089**	**0.069**	**0.065**	**0.081**	**0.189**					
D9	0.051	**0.129**	**0.064**	0.059	0.037	0.038	**0.098**	**0.101**				

	F1	F2	F3	F4	F5	F6	F7	F8	F9	F10		
F1												
F2	0.020											
F3	0.090	0.099										
F4	**0.142**	**0.075**	**0.129**									
F5	0.050	0.070	0.121	**0.114**								
F6	0.019	0.036	**0.128**	**0.102**	0.001							
F7	0.051	0.037	**0.187**	**0.148**	0.044	**0.082**						
F8	**0.244**	**0.223**	**0.350**	**0.224**	**0.182**	**0.184**	**0.276**					
F9	0.082	0.093	**0.244**	**0.167**	0.053	0.072	0.082	**0.248**				
F10	0.100	**0.105**	**0.231**	**0.155**	0.070	0.101	0.073	**0.282**	−0.008			

1 Numbers in bold indicate significance in genepop exact test for differentiation.

Results of the AMOVA suggested that the percentages of total genetic variation among locations (as opposed to within locations) were as follows: University of Miami (earliest colonized), 3%; Everglades, 9%; Fort De Soto, 13%; and F.I.T. (most recently colonized), 17%. These results reflect a relative lack of differentiation among locations at Miami and highest differentiation at F.I.T., the most recently colonized site. There was no evidence for isolation by distance (IBD) at any of the four sites according to Mantel tests (*r*_M_ = −0.091–0.196; *P* = 0.09–0.22), most likely because populations are not at equilibrium. At Fort De Soto, there appeared to be substantial differentiation between the campground locations and the other locations, but within each group of locations there was no evidence for isolation by distance. There was a positive spatial autocorrelation at all sites except Miami. In the Everglades, there was positive autocorrelation (*P*<0.05) over distances of 70 meters (*r* = 0.102; *r* at lesser distances ranged from 0.077–0.095). At Fort De Soto, there was positive autocorrelation over distances of 80 meters (*r* = 0.455; *r* at lesser distances ranged from 0.089–0.220). At F.I.T., there was positive autocorrelation over distances of 70 meters (*r* = 0.079; *r* at lesser distances ranged from 0.057–0.173).

**Figure 1:**
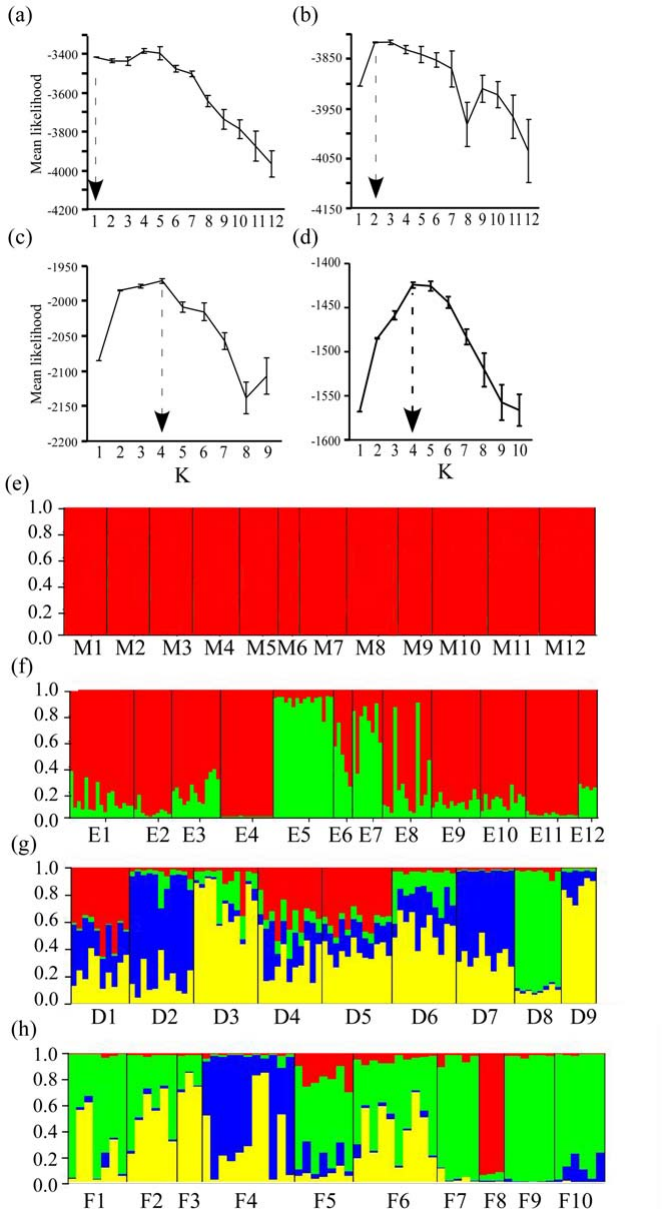
Population structure within four sites differing in arrival time. structure [41] analyses show mean (±SE) likelihood at each K over 10 runs, and dotted arrows show the probable true value of K for each group of locations: (A) Miami, (B) Everglades, (C) Forst De Soto, and (D) F.I.T. Proportional membership of each individual gecko (thin vertical line) to each cluster, represented by different colors, for all four sites: (E) Miami, (F) Everglades, (G) Fort De Soto, and (H) F.I.T. Black vertical lines separate individuals by buildings indicated below each group.

**Figure 2:**
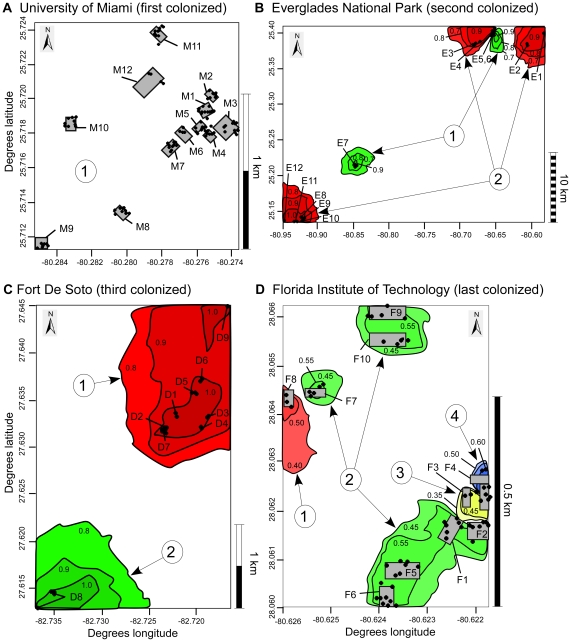
Maps of building locations and population clusters at four sites. Contour lines indicate probability of membership in a cluster determined by geneland. Exact sampling locations for each individual (black dots) at (A) University of Miami, (B) Everglades, (C) Fort De Soto, and (D) F.I.T. Each population cluster is indicated by circled numbers (no structure was detected in Miami). Grey boxes represent approximate building locations and perimeters. Letter-number building codes correspond to those in Table 1.

At the earliest colonized site, the University of Miami, there were no significant genetic clusters detected by the Bayesian clustering method (structure; Fig. 1a) or by the spatial Bayesian clustering method (geneland; Fig. 2a). In the Everglades, both methods detected two clusters, with locations E5, E6, and E7 comprising one cluster, and all other locations comprising the second cluster (Fig. 1b, 1f, 2b). At Fort De Soto, structure revealed significant support for at least two clusters by one ad hoc method [48], but evidence for four clusters is apparent (Figs. 1c, 1g), as evidenced by the spatial segregation of assigned populations [40], [41]. The geneland analysis also found two clusters (Fig. 2c). At F.I.T., structure identified four clusters (Fig. 1d, 1h), and the clusters were the same as the four identified by geneland (Fig. 2d).

In our tests for migration-drift equilibrium, there was support for the gene flow model in the three sites occupied for the longest period of time, which suggests locations within these sites are nearer to equilibrium: Miami (*P* gene flow = 0.57, Bayes factor 1.31), the Everglades (*P* gene flow = 0.72, Bayes factor 2.62), and Fort De Soto (*P* gene flow = 0.99, Bayes factor 86.38). However, F.I.T., showed evidence for the drift model, which suggests locations within this site have been more recently colonized (*P* drift = 0.66, Bayes factor 1.91).

### Genetic Diversity

**Figure 3:**
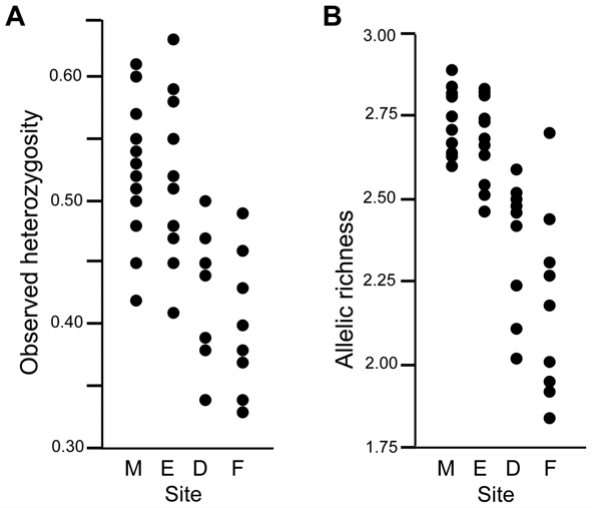
Genetic diversity among sites and locations differs according to colonization sequence. Observed heterozygosity (A) and allelic richness (B) at all 43 locations from four sites.

Pronounced genetic structure that arises rapidly is likely the product of genetic drift, which predicts that recently colonized sites should be less genetically diverse. Locations at the two most recently colonized sites, Fort De Soto and F.I.T., had significantly lower allelic richness than those at the other two sites, the University of Miami and Everglades National Park (Table 3; Fig. 3). However, when locations within sites were pooled, there were no significant differences in allelic richness among sites. Locations at Fort De Soto and F.I.T. had lower observed heterozygosity than other locations nearer the point of origin (Table 3; Fig. 3). Together, these results suggest that more recently colonized locations have lower genetic diversity than longer established locations. Three locations (M9, E3, and F8) showed heterozygosity excess indicative of recent bottlenecks according to the bottleneck program. One location in the Everglades (E11) had heterozygosity deficit according to the program, and this could be due to recent admixture occurring at this location.

**Table 3:** Differences in genetic diversity among sites.^1^

	Miami	Everglades	Fort De Soto	F.I.T.
Miami	*	NS	<0.001	0.002
Everglades	NS	*	0.003	0.011
Fort De Soto	0.003	0.013	*	NS
F.I.T.	<0.001	<0.001	NS	*

1 Numbers reflect P-values from tests of differences in genetic variation between locations in terms of allelic richness per location (below diagonal) and observed heterozygosity per location (above diagonal). NS corresponds to *P*>0.05. Directionality of differences corresponds to time since colonization (e.g. Miami has higher allelic richness and higher heterozygosity than Fort De Soto, etc.).

## Discussion

**Figure 4:**
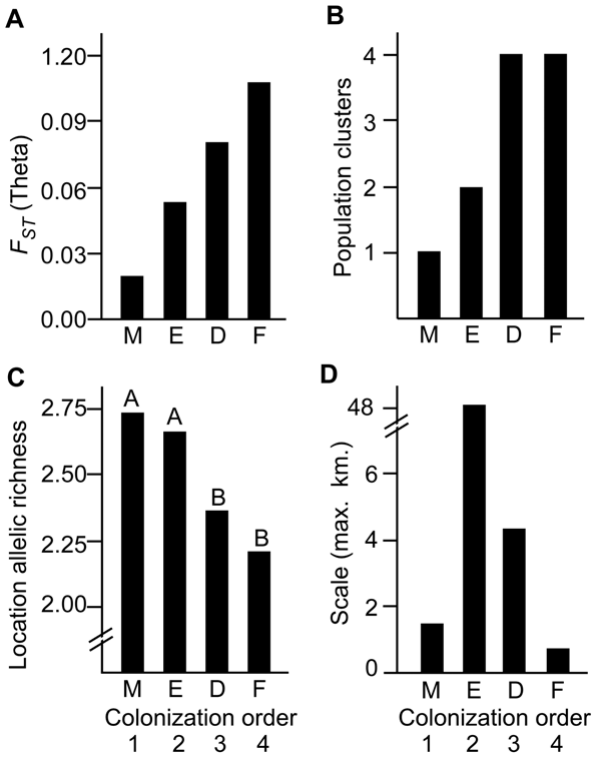
Genetic diversity decreases and population structure increases toward the leading edge of invasion. (A) *F_st_* (*θ*), (B) number of clusters according to structure, (C) allelic richness, and (D) geographic scale for 43 locations within four sites. Letters above the bars correspond to significance groups.

This is one of very few studies to empirically test theoretical predictions that genetic structure can arise at a fine spatial scale during range expansion. Of the 43 populations of introduced *H. mabouia* surveyed from small habitat patches, those closer to the leading edge of range expansion showed pronounced genetic structure at surprisingly small spatial scales (<100 m), while other populations colonized earlier near the point of introduction showed little evidence of genetic structure (Fig. 4). The genetic structure near the leading edge is likely a consequence of colonization and genetic drift, as evidenced by the lower levels of genetic diversity in recently colonized locations, and higher levels of genetic diversity in locations occupied for longer periods of time (Fig. 4). These findings support theoretical predictions that populations at the leading edge of range expansion should exhibit marked genetic structuring due to limited dispersal during colonization [2], [3], [4].

### Population Structure and Gene Flow

Several lines of evidence suggest that gene flow during colonization was limited, indicating fine-scale dispersal limitation in *H. mabouia*. Locations at all sites except Miami showed evidence of significant population structure, and Bayesian clustering analyses supported the conclusion that gene flow was limited among locations especially at Fort De Soto and F.I.T. The significant spatial autocorrelations also suggest that gene flow was limited even within buildings at three more recently colonized sites, and we suspect this is a signature mostly of non-equilibrium colonization processes. Miami is likely closer to migration-drift equilibrium, but it did not show evidence of spatial autocorrelation, possibly because at later stages of the invasion process, there are many more possible sources of immigrants and the scales considered may be too small to detect a spatial autocorrelation.

The overall patterns of genetic structure among sites appear to be most closely related to time since colonization (Fig. 4). Although other factors may be present and may differ among sites, they do not account for the observed patterns of genetic structure. For instance, Miami and F.I.T. are both college campuses with very similar intervening habitat, but they lie on opposite ends of the spectrum of genetic structure. F.I.T. has smaller inter-building distances than Miami, and yet it still has more genetic structure. The site with the largest distances among buildings (Everglades) had genetically indistinguishable populations at the geographical extremes of the site, but it also had fine-scale genetic differences among some nearby buildings (Fig. 2). In this case we can see both limited dispersal and genetic structure on a small scale, as well as evidence of very long-distance colonization that is likely facilitated by human movements. Long-distance colonizations may help to establish initial genetic differences within sites. At F.I.T., location F4 was a construction site where building materials were being brought in, and F4 was genetically very different than the adjacent location F3 (Fig. 2). At Fort De Soto, location D8 was a pier with high visitor traffic, and it was genetically distinct in structural analyses. Over time, we expect that genetic structure will be reduced by both small-scale natural dispersal and continued long-distance, human aided dispersal.

### Genetic Diversity

The evidence of reduced genetic diversity in more recently colonized populations (Table 3; Fig. 3) is consistent with the notion that genetic drift plays a role at the leading edge of range expansion [3], [4]. Although expected heterozygosity values were relatively high at all locations, observed heterozygosities were significantly lower at locations within Fort De Soto and F.I.T. than at Miami and the Everglades. This difference likely indicates initially low population sizes, inbreeding, and drift in several different newly colonized habitat patches within each of the more recently invaded sites. The smaller sample sizes from the leading edge of invasion reflect lower density patches that are farther from carrying capacity, as expected, but the methods used to reveal the patterns are not sensitive to differences in sample size, so our results are not attributable to sample size differences. The general lack of evidence for genetic bottlenecks may be due to small sample sizes from each location, the limited duration or recent nature of the bottleneck [46], [47], high population growth [49] or low levels of subsequent immigration [50].

One departure from expectations was that tests for migration-drift equilibrium suggested Fort De Soto, with the second most recently colonized locations, and significant substructure among locations, had the highest overall levels of gene flow. The genetic similarity is confined to bath houses and we can think of two possible explanations for high gene flow. The campground matrix is scrub with a high density of palm trees that geckos can use and may facilitate natural movements between structures. Alternatively, daily rounds are made with trucks pulled up alongside structures to deliver supplies and collect waste, and this may augment gecko movements among this subset of locations.

Interestingly, allelic richness values pooled among locations did not differ among the four sites. This suggests that while sites did not differ in their overall genetic diversity, they differed in how genetic diversity was distributed among individual locations within sites. Miami and the Everglades appear to be nearer to migration-drift equilibrium because locations at these sites contain a greater proportion of the total allelic richness. At Fort De Soto and F.I.T., however, gene flow among buildings appears to be low perhaps because not enough time has elapsed to homogenize alleles among locations. At these short time scales, ecological and demographic factors likely come into play. A period of time is expected where emigration is bound to be low after colonization but before a location to reaches carrying capacity. Longitudinal studies [24], [25] suggest this period can take somewhere on the order of five to ten years, which is roughly equivalent to the same number of generations.

### Conclusions

Genetic processes of range expansion may be important for understanding natural range expansions, biological invasions, and tracking of habitat shifts due to climate change [51], [52]. Even transient genetic structure during range expansion may be important because it may affect the ability of populations to adapt to local conditions. Genetic structure at the leading edge of range expansion also sets the stage for possible mutation surfing, where rare mutations can be propagated by serial colonization at an expanding range front [7]. Although we have only demonstrated the existence of genetic structure at presumably neutral marker loci, this study supports theoretical predictions by showing that such genetic structuring can occur on very short time scales in nature. While some studies have shown that dispersal and gene flow may be limited over small distances [12], [16], [52], [53], most studies of range expansion have focused on larger landscape patterns [13], [14]. In this study we found significant genetic structuring at spatial scales as small as tens of meters. Although the patterns we revealed are consistent with those predicted by theory, we are left with some level of uncertainty regarding the exact processes that have caused these patterns. It is our hope that these results will prompt researchers to study range expansion in other systems at a finer scale than is normally considered, and eventually reveal how small scale processes affect trait evolution and adaptation during range expansion.

## Supporting Information

Table S1 Location codes and corresponding building names for four sampling sites.
(DOCX)
