# Supplementary material for: Fine-Scale Genetic Structure Arises during Range Expansion of an Invasive Gecko
Source: PLoS One. 2011 Oct 28;6(10):e26258. doi: 10.1371/journal.pone.0026258 (PMC3203895; doi:10.1371/journal.pone.0026258)
Supplement: Table S1 — Location codes and corresponding building names for four sampling sites. (DOCX) [file pone.0026258.s001.docx]

**Table S1. (supplementary) Location codes and corresponding building names for four sampling sites.**

| **University of Miami** | |
| --- | --- |
| M1 | Lowe Art Museum |
| M2 | Behavioral Medicine Building |
| M3 | Mahoney Residential College |
| M4 | Baptist Ministry |
| M5 | Wesley Foundation |
| M6 | Student Services Center |
| M7 | Air Force ROTC |
| M8 | Sculpture Studio |
| M9 | Founders Hall/Alumni Relations |
| M10 | St. Augustine Catholic Church |
| M11 | Building L-1 |
| M12 | Richter Library |
| **Everglades National Park** | |
| E1 | Ernest F. Coe Visitor Center |
| E2 | Royal Palm Visitor Center |
| E3 | Daniel Beard Center |
| E4 | Bill Robertson/Fire Cache |
| E5 | Long Pine Key Bathroom |
| E6 | Long Pine Key Entrance |
| E7 | West Lake Bathroom |
| E8 | Gas Station |
| E9 | Marina Bathrooms |
| E10 | Marina Store |
| E11 | Flamingo Visitor Center |
| E12 | Campground Bathrooms |
| **Fort De Soto Campground** | |
| D1 | Campground Bathroom 1 |
| D2 | Campground Bathroom 2 |
| D3 | Campground Bathroom 3 |
| D4 | Campground Bathroom 4 |
| D5 | Campground Bathroom 6 |
| D6 | Campground Bathroom 7 |
| D7 | Campground Dayhouse |
| D8 | Gulf Pier Bathroom |
| D9 | Boat Ramp Bathroom |
| **Florida Institute of Technology** | |
| F1 | Allen S. Henry Building |
| F2 | College of Business |
| F3 | Fish Biology |
| F4 | Ecophysiology |
| F5 | Aerospace Lab |
| F6 | All Faiths Center |
| F7 | Holzer Health Center |
| F8 | Community Psych. Services |
| F9 | Miller Quad North |
| F10 | Miller Quad South |
